# Prophylactic management of postpartum haemorrhage in the third stage of labour: an overview of systematic reviews

**DOI:** 10.1186/s13643-018-0817-3

**Published:** 2018-10-11

**Authors:** Yuko Masuzawa, Yaeko Kataoka, Kana Fujii, Satomi Inoue

**Affiliations:** 1 Graduate School of Nursing Science, St. Luke’s International University, 10-1, Akashi-cho, Chuo-ku, Tokyo, 104-0044 Japan; 2 St. Luke’s International University, 10-1, Akashi-cho, Chuo-ku, Tokyo, 104-0044 Japan

**Keywords:** Postpartum haemorrhage, Third-stage labour, Prevention, Randomized controlled trial, Systematic review, Overview of systematic review

## Abstract

**Background:**

Postpartum haemorrhage is a direct cause of maternal death worldwide and usually occurs during the third stage of labour. Most women receive some type of prophylactic management, which may include pharmacological or non-pharmacological interventions. The objective of this study was to summarize systematic reviews that assessed the effects of postpartum haemorrhage prophylactic management during the third stage of labour.

**Methods:**

We applied the guidelines for conducting an overview of reviews from the Cochrane Handbook for Systematic Reviews of Interventions. We searched MEDLINE, EMBASE, and the Cochrane Database of Systematic Reviews to identify all relevant systematic reviews of randomized controlled trials of prophylactic management of postpartum haemorrhage in the third stage of labour compared with no treatment, placebo, or another management technique. Two review authors independently extracted data and assessed methodological quality using a measurement tool to assess reviews and quality of evidence using the Grades of Recommendation, Assessment, Development, and Evaluation for primary outcomes, summarizing results narratively.

**Results:**

We identified 29 systematic reviews: 18 Cochrane and 11 non-Cochrane. Cochrane systematic reviews were high quality, while the quality of non-Cochrane systematic reviews varied. The following techniques suggested effective, third-stage interventions to reduce the incidence of severe postpartum haemorrhage: active management of the third stage of labour compared to physiological management, active management compared to expectant management, administration of oxytocin compared to placebo, and use of tranexamic acid compared to placebo. The following third-stage management approaches reduced the need for blood transfusion: active management compared to physiological management, active management compared to expectant management, oral misoprostol compared to placebo, and tranexamic acid compared to placebo.

**Conclusions:**

No effective prophylactic management techniques were identified for maternal mortality. Most methods of effective prophylactic management of postpartum haemorrhage were supported by evidence; however, they were limited to low- or moderate-quality evidence, and high-quality studies are therefore needed. Outcome measures of the included systematic reviews varied. It is recommended that outcome measures in preventive postpartum haemorrhage intervention trials align with the World Health Organization guidelines.

**Systematic review registration:**

PROSPERO: CRD42016049220.

**Electronic supplementary material:**

The online version of this article (10.1186/s13643-018-0817-3) contains supplementary material, which is available to authorized users.

## Background

### Description of the condition

Maternal mortality is an indicator of women’s health [1]. The primary cause of maternal death in both developing and developed countries is postpartum haemorrhage (PPH) [2, 3], representing 19.7% of maternal deaths worldwide [4]. PPH is defined as blood loss ≥ 500 mL within the first 24 h after delivery [5]. The prevalence of PPH (blood loss ≥ 500 mL) is approximately 6.0–10.0%, and the prevalence of severe PPH (blood loss ≥ 1000 mL) is approximately 1.8–3.0% in any type of delivery but varies by region in the world [6]. Representing a significant medical threat worldwide, effective strategies for the prevention of PPH are essential to decrease maternal mortality rates.

The period during delivery of the baby and placenta is defined as the third stage of labour and is a critical time for the occurrence of PPH [7]. After the third stage of labour, haemostasis processes are activated. Contraction of the uterine muscles is the primary physiological process for postpartum haemostasis and results from the actions of oxytocin and prostaglandins. The uterine smooth muscles are spiral structures that extend in all directions. The spiral vessels of the uterus are compressed by the contractions of uterine muscles, which lead to decreased blood flow [8]. Moreover, blood clotting and fibrinolytic factors increase in pregnant women [9]. The failure of these physiological mechanisms during postpartum haemostasis is one of the causes of PPH. Events that may influence PPH include uterine atony, cervical or vaginal lacerations, retention of the placenta, and coagulation disorders [10, 11]. Among these, the most prominent cause of PPH is uterine atony (34.0%) [12]. PPH has 19 identified risk factors: age ≥ 35 years [13], body mass index ≥ 30 kg/m^2^ [14], Pacific island or Asian ethnicity [14, 15], parity of three or more [13], primiparity [12, 15, 16], multiple birth [12, 15, 17], history of PPH [15], hypertensive disorders [14], pre-eclampsia [14, 15], placenta previa [14, 17], placental abruption [17], retained placenta [16], induction of labour [13–15], prolonged labour [12, 15, 16], obstructed labour [12], episiotomy [14, 15], instrumental labour [14, 15], caesarean section [13], and gestational age at delivery < 37 weeks [13, 17].

### Description of the intervention

Given that PPH usually occurs during and after the third stage of labour [6], primary guidelines recommend active management of this stage. The main component of effective PPH prophylactic management is the administration of uterotonics [5, 10, 18–20]. Active management is a set of prophylactic interventions consisting of the following components: administration of uterotonics after delivery, early umbilical cord clamping, controlled cord traction for earlier delivery of the placenta, and in certain cases, uterine massage [5].

In contrast with active management, expectant and physiological managements are “hands-off” techniques involving no administration of prophylactic uterotonic agents and delivery of the placenta only through maternal efforts [5]. The World Health Organization (WHO) guideline for preventing PPH recommend the following interventions: use of uterotonics during the third stage of labour for all births, use of oxytocin (10 IU) as the uterotonic drug, controlled umbilical cord clamping in settings where skilled birth attendants are available, and late cord clamping [5]. Moreover, the National Institute for Health and Care Excellence’s guidelines were revised in 2014, including a change in the definition of active management of the third stage of labour. The ideal timing of cord clamping, as one component of active management, was changed from early to late, as evidence indicated that late cord clamping did not negatively impact maternal outcomes and had benefits for the neonate [18].

The effectiveness of some prophylactic management techniques for PPH in the third stage of labour has been evaluated in Cochrane systematic reviews. Active management of the third stage of labour was evaluated in comparison with expectant management [21]. Early cord clamping plus controlled cord traction, as one component of active management, is believed to prevent retained placenta and a prolonged third stage of labour. The effectiveness of the timing of cord clamping [22] and controlled cord traction [23] has been recently evaluated.

Other prophylactic interventions were considered in this overview, including use of uterotonic drugs, use of hemostatic agents and uterine massage. For uterotonic drugs, the following were considered as representative methods of augmenting uterine contractions: oxytocin [24–29], prostaglandin [30–32], and ergot alkaloid [33]. Oxytocin is a naturally occurring hormone that stimulates uterine contractions [34] and is commonly used as a uterotonic. The half-life of oxytocin is short (4–7 min) [35]; therefore, both repeated doses and continuous infusion are acceptable [20]. Prostaglandin is also a naturally occurring hormone; misoprostol, a prostaglandin E1 analogue, can be used orally, sublingually, vaginally, or rectally [36]. Furthermore, misoprostol has mild side effects, such as shivering and pyrexia [37]. Ergot alkaloids act to contract the myometrium through calcium channel mechanisms; however, this also increases the incidence of side effects such as hypertension [38]. A survey examining the use of prophylactic uterotonic agents in 28 countries noted that 95.3% of deliveries used prophylactic uterotonics for the prevention of PPH, and the most commonly used uterotonic agent was oxytocin [13].

### Why it is important to do this review

Many prophylactic management techniques for PPH in the third stage of labour have been evaluated in systematic reviews of randomized controlled trials (RCTs). The extant systematic reviews of evidence from RCTs regarding prophylactic management of PPH in the third stage of labour have never been summarized. Furthermore, many clinical guidelines for preventing PPH [18–20] were published and are reflected in these systematic reviews. This overview will allow the many readers (such as clinicians, midwives, policy makers, and consumers) to quickly assess a range of evidence about prophylactic management techniques for PPH and utilize this information for making decisions. Regarding the application to research, through summarizing the effectiveness of interventions by outcomes, this overview will also provide a set of outcome measures that are clinically meaningful that can be applied to future studies.

### Objectives

The objective of this overview was to:

- provide a narrative summary of systematic reviews of RCTs;
- provide the effectiveness of prophylactic management of PPH during the third stage of labour of any type of delivery (vaginal or caesarean section) in terms of outcome measures including maternal mortality, blood loss greater than 1000 mL, and use of blood transfusion.

## Methods

In this overview, we applied the guidelines for conducting an overview of reviews from the Cochrane Handbook for Systematic Reviews of Interventions [39] and adhered to the systematic reporting guidelines of the preferred reporting items for systematic reviews and meta-analysis (PRISMA) statement [40]. The PRISMA checklist is shown in Additional file 1. Our review protocol was registered with the International Prospective Register of Systematic Reviews (PROSPERO) (CRD42016049220).

### Criteria for considering included review

#### Type of reviews

In this overview, we included published systematic reviews of RCTs in which prophylactic management of PPH in the third stage of labour was administered after delivery. We excluded reviews that were not systematic reviews of RCTs and those that were only abstracts. When the identified Cochrane review was an updated review with a previous version, we also excluded the previous version and only included the updated version.

#### Type of participants

The review subjects were women who delivered vaginally or by caesarean section. We recognize that the risk of PPH for vaginal delivery or caesarean section may vary, but we included both delivery methods because some of the reviews presented their results regardless of the mode of delivery.

#### Type of intervention and comparisons

We included any prophylactic managements of PPH in the third stage of labour and divided them into the following subgroups.

##### Pharmacological interventions

- Active management of the third stage of labour
- Oxytocin
- Prostaglandin
- Ergot alkaloids
- Tranexamic acid

##### Non-pharmacological interventions

- Early umbilical cord clamping
- Controlled cord traction
- Uterine massage

We excluded non-prophylactic management. We compared these interventions with placebo, no treatment, contrasting interventions, or other interventions.

#### Type of outcomes

We searched for the three critical outcomes proposed in the WHO’s recommendations for the prevention and treatment of postpartum haemorrhage guidelines [5].

- Maternal mortality
- Blood loss greater than 1000 mL
- Use of blood transfusion

### Search strategy

A comprehensive search was conducted for relevant reviews published in any language in MEDLINE (via EBSCO, 11 October 2016), EMBASE (1980 to 11 October 2016), Cochrane Database of Systematic Reviews (issue 10 of 12 October 2016), and Database of Abstracts of Reviews of Effect (Cochrane library issue 2 of 4 April 2015), using the search terms “postpartum haemorrhage” and “prevention.” Systematic review search filters in clinical evidence [41] were used to search MEDLINE and EMBASE. The search strategy is detailed in Additional file 2.

### Selection of systematic reviews

Two authors (YY, KF) independently assessed all potential systematic reviews that resulted from our search strategy for inclusion in the present review. We resolved any disagreement through discussion and/or by consulting the third author (YK).

### Data extraction

A predefined form was used for data extraction, which included the following sections: study design, participants, experimental intervention, comparison intervention, outcomes, quality of the review, and pooled effect sizes for main outcome measures in metanalyses. When an included review did not identify the number of outcome events or was not meta-analysed, we verified the primary sources included in the review and then performed meta-analysis ourselves using Review Manager 5.3 [42]. We assessed each study’s statistical heterogeneity using *I*^2^ statistics. Where heterogeneity was observed (*I*^2^ > 50%), we conducted a random-effects model for combining data. A fixed-effect model was used if the heterogeneity indicated non-importance (*I*^2^ < 50%). We presented a risk ratio with 95% confidence intervals (CI) about dichotomous data.

Two review authors (YM, SI) independently extracted data from the reviews using the data extraction form. We entered data into the Review Manager software 5.3 [42] and GRADEpro GDT software [43] and verified accuracy.

### Quality of evidence in included reviews

Quality of evidence in the included reviews was examined using the Grades of Recommendation, Assessment, Development and Evaluation (GRADE) approach [44] for outcomes. The GRADE approach is a system that evaluates quality of evidence, which is assessed on a 4-point scale (“very low,” “low,” “moderate,” and “high”) in five domains: (1) study limitations, (2) inconsistency of results, (3) indirectness of evidence, (4) imprecision, and (5) publication bias [44]. We assessed “study limitation” by using the approach of the Cochrane risk of bias tool [39]. If the original study did not assess the risk of bias, we assessed the risk of bias on low, high, or unclear for: (1) selection biases, (2) performance bias, (3) detection bias, (4) attrition bias, (5) reporting bias, and (6) other potential sources of bias [39]. When we assessed the domain of the “imprecision” of the GRADE approach, we focused on the 95% CI around the difference in effect between intervention and comparison. We concluded that the imprecision was not serious when: (1) the 95% CI did not include the RR of 1.0 and included appreciable benefit or harm (RR of under 0.75 or over 1.25); and (2) the total event number and the optimal information size were enough [45]. We used the GRADEpro GDT software’s [43] “summary of findings” tables for each outcome.

### Assessment of methodological quality of included reviews

The review authors (YM, KF, YK) independently assessed the quality of evidence and methodological quality of the included reviews using the instrument: a measurement tool to assess reviews (AMSTAR) [46]. The AMSTAR tool [46] consists of 11 criteria for measuring the methodological quality of systematic reviews, which is determined by a questionnaire with 11 items that asks reviewers to answer “yes,” “no,” “cannot answer,” or “not applicable.” We resolved discrepancies through discussion.

### Data synthesis

We provided a narrative summary of the individual review results for each outcome displayed in tables and figures that included characteristics of each review, AMSTAR ratings and outcomes using GRADE. Although we planned to present the data divided by the mode of delivery, almost all original reviews including vaginal and caesarean section deliveries presented the data together, not separately. Therefore, we did not provide the review result for each outcome divided by the delivery mode.

## Results

### Description of included reviews

**Fig. 1:**
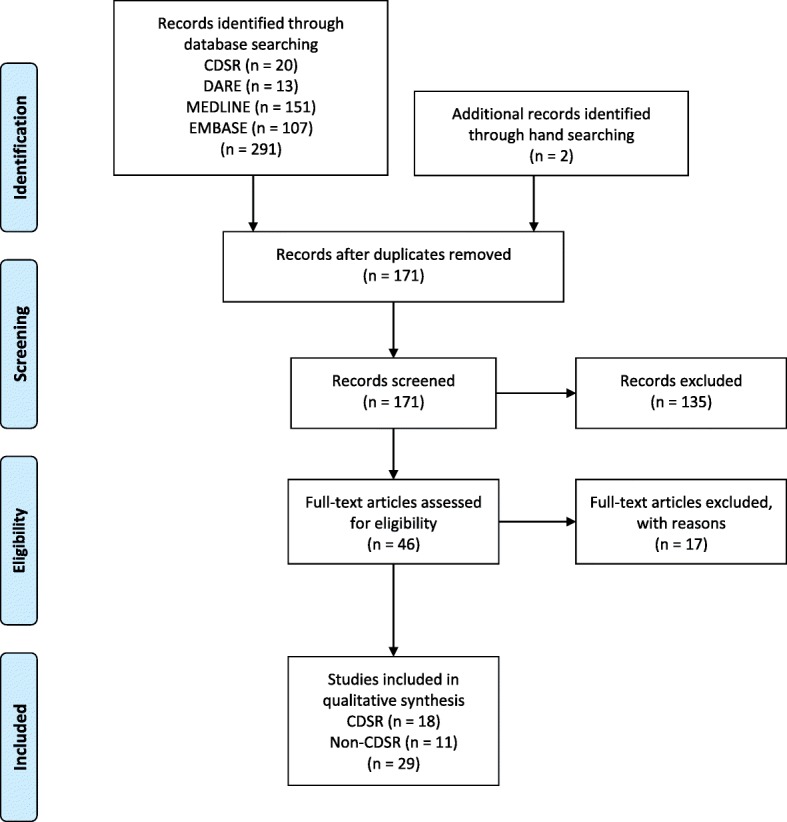
Study flow diagram using the PRISMA 2009 flow diagram

Figure 1 is a flow diagram of the selection process. A total of 291 studies were identified from the database search. After removing duplicates, 171 studies remained. A total of 135 titles and abstracts were excluded because they were not systematic reviews or did not examine prophylactic management of PPH. There were 46 full-text studies remaining; of these, 17 were excluded because they did not include prophylactic management, were not systematic reviews of RCTs, or were only abstracts. The excluded studies list is detailed in Additional file 3. A final sample of 29 studies met the inclusion criteria.

Of the 29 systematic reviews, 18 Cochrane systematic reviews and 11 non-Cochrane systematic reviews were analysed: five studies examining active management of the third stage of labour [21, 23, 47–49], eight examining the use of oxytocin [24–29, 50, 51], seven examining the use of prostaglandin [30–32, 52–55], one examining the use of ergot alkaloids [33], five examining tranexamic acid [56–60], one examining timing of umbilical cord clamping [22], one examining uterine massage [61], and one examining breastfeeding or nipple stimulation [62]. The participants in these reviews had undergone caesarean or vaginal deliveries. Table 1 shows the characteristics of these reviews.

**Table 1:** Characteristics of included systematic reviews

Review title	Date of search	No. studies included	Population	Intervention	Comparison intervention	Outcomes for which data were reported
Active management						
Cochrane review						
“Fundal pressure versus controlled cord traction as part of the active management of the third stage of labour” (Peña-Martí, 2007 [49])	August 2010	No RCTs	N/A	Fundal pressure with routine administration of a uterotonic drug and early cord clamping	Controlled cord traction with routine administration of a uterotonic drug and early cord clamping	Empty review
“Active versus expectant management for women in the third stage of labour” (Begley, 2015 [21])	30 September 2014	7 RCTs	8247 women (vaginal birth at > 24 weeks’ gestation)	Active management of the third stage of labour	Expectant management of the third stage of labour	• Severe PPH (≥ 1000 mL) • Maternal blood transfusion • PPH (≥ 500 mL) • Therapeutic uterotonics
“Controlled cord traction for the third stage of labour” (Hofmeyr, 2015 [23])	29 January 2014	3 RCTs	28,049 women (vaginal birth)	Controlled cord traction with uterotonics	No controlled cord traction with uterotonics	• Maternal death • Severe PPH (≥ 1000 mL) • Maternal blood transfusion • PPH (≥ 500 mL) • Therapeutic uterotonics
Non-Cochrane review						
“Preventing postpartum hemorrhage in low-resource settings” (McCormick, 2002 [48])	September 2001	3 RCTs	4855 women (vaginal birth)	Active management of the third stage of labour	Physiologic management	• Severe PPH (≥ 1000 mL) • Maternal blood transfusion • PPH (≥ 500 mL) • Therapeutic uterotonics
“Active management of the third stage of labor with and without controlled cord traction: A systematic review and meta-analysis of randomized controlled trials” (Du, 2014 [47])	30 October 2013	5 RCTs	30,532 women (vaginal birth)	Controlled cord traction	Hands-off Physiological expulsion of placenta	• Maternal death • Severe PPH (≥ 1000 mL) • Maternal blood transfusion • PPH (≥ 500 mL) • Therapeutic uterotonics
Pharmacological management (oxytocin)						
Cochrane review						
“Prophylactic ergometrine-oxytocin versus oxytocin for the third stage of labour” (McDonald, 2004 [24])	30 April 2007	6 RCTs	9332 women (vaginal birth)	Ergometrin-eoxytocin	Oxytocin	• Severe PPH (≥ 1000 mL) • Maternal blood transfusion • PPH (≥ 500 mL) • Therapeutic uterotonics
“Timing of prophylactic uterotonics for the third stage of labour after vaginal birth” (Soltani, 2010 [27])	September 2009	3 RCTs	1671 women (vaginal birth)	Intramuscularly or infusion of oxytocin (10 or 20 units), at delivery of the baby’s shoulder, or after the second stage of labour	Intramuscularly or infusion of oxytocin (10 or 20 units), after the birth of placenta	• Severe PPH (≥ 1000 mL) • Maternal blood transfusion • PPH (≥ 500 mL) • Therapeutic uterotonics
“Umbilical vein injection for the routine management of third stage of labour” (Mori, 2012 [25])	31 January 2012	9 RCTs	1118 women (vaginal birth)	Normal saline or uterotonic drugs, or both, via the umbilical cord	Other alternatives (similar agents IV or IM or no injection/placebo)	• Maternal blood transfusion
“Intramuscular versus intravenous prophylactic oxytocin for the third stage of labour” (Oladapo, 2012 [26])	31 December 2011	No RCTs	N/A	Intramuscular oxytocin	Intravenous oxytocin	Empty review
“Carbetocin for preventing postpartum haemorrhage” (Su, 2012 [28])	1 March 2011	11 RCTs	2635 women (caesarean or vaginal birth)	Oxytocin agonist (carbetocin)	Other uterotonic agents or with placebo or no treatment	• Severe PPH (≥ 1000 mL) • Maternal blood transfusion • PPH (≥ 500 mL) • Therapeutic uterotonics
“Prophylactic oxytocin for the third stage of labour to prevent postpartum haemorrhage” (Westhoff, 2013 [29])	31 May 2013	20 RCTs	10,806 women (vaginal birth)	Prophylactic oxytocin	Placebo or ergot alkaloids	• Maternal death • Severe PPH (≥ 1000 mL) • Maternal blood transfusion • PPH (≥ 500 mL) • Therapeutic uterotonics
				Oxytocin plus ergometrine	Ergot alkaloids	
“Oxytocin for preventing postpartum haemorrhage (PPH) in non-facility birth settings” (Pantoja, 2016 [51])	12 November 2015	1 RCT	5919 women (vaginal birth)	Prophylactic oxytocin (any strategy)	No intervention or other uterotonics	• Maternal death • Severe PPH (≥ 1000 mL) • PPH (≥ 500 mL)
Non-Cochrane review						
“Carbetocin for the prevention of postpartum hemorrhage: a systematic review and meta-analysis of randomized controlled trials” (Jin, 2016 [50])	September 2013	12 RCTs	2975 women (caesarean or vaginal birth)	Carbetocin	Other uterotinic agents	• Severe PPH (≥ 1000 mL) • Maternal blood transfusion • Therapeutic uterotonics
Pharmacological management (prostaglandin)						
Cochrane review						
“Advance misoprostol distribution for preventing and treating postpartum haemorrhage” (Oladapo, 2012 [31])	5 October 2011	No RCTs	N/A	Advance misoprostol distribution	Usual care for PPH prevention or treatment	Empty review
“Prostaglandins for preventing postpartum haemorrhage” (Tunçalp, 2012 [32])	7 January 2011	72 RCTs	52,678 women (caesarean or vaginal birth)	Prostaglandin agent in the third stage of labour	Another uterotonic or no prophylactic uterotonic (nothing or placebo)	• Severe PPH (≥ 1000 mL) • Maternal blood transfusion • PPH (≥ 500 mL) • Therapeutic uterotonics
“Postpartum misoprostol for preventing maternal mortality and morbidity” (Hofmeyr, 2013 [30])	11 January 2013	78 RCTs	59,216 women (caesarean or vaginal birth at > 24 weeks’ gestation)	Misoprostol	Placebo/no treatment or other uterotonics	• Maternal death
Non-Cochrane review						
“Misoprostol use during the third stage of labor” (Joy, 2003 [53])	January 1996 to May 2002	17 RCTs	28,170 women (caesarean or vaginal birth)	Misoprostol	Placebo or other uterotonics	• Severe PPH (≥ 1000 mL) • PPH (≥ 500 mL) • Therapeutic uterotonics
“Misoprostol in preventing postpartum hemorrhage: a meta-analysis” (Langenbach, 2006 [54])	May 2005	22 RCTs	30,017 women (caesarean or vaginal birth)	Misoprostol	Placebo or oxytocics	• Severe PPH (≥ 1000 mL) • PPH (≥ 500 mL) • Therapeutic uterotonics
“Misoprostol to prevent and treat postpartum haemorrhage: a systematic review and meta-analysis of maternal deaths and dose-related effects” (Hofmeyr, 2009 [52])	February 2007	46 RCTs	More than 40,000 women (caesarean or vaginal birth)	Misoprostol	Placebo/other uterotonics	• Maternal death • Severe PPH (≥ 1000 mL) • PPH (≥ 500 mL)
Misoprostol for prevention and treatment of postpartum haemorrhage: a systematic review (Olefile, 2013 [55])	Unclear	3 RCTs	2346 women (vaginal birth)	Misoprostol	Placebo for the prevention and treatment of PPH	• Severe PPH (≥ 1000 mL) • Maternal blood transfusion • PPH (≥ 500 mL) • Therapeutic uterotonics
Pharmacological management (ergot alkaloid)						
Cochrane review						
“Prophylactic use of ergot alkaloids in the third stage of labour” (Liabsuetrakul, 2007 [33])	30 April 2011	6 RCTs	1996 women (vaginal birth)	Any ergot alkaloid given prophylactically, by whatever route or timing of administration	No uterotonic agents	• Severe PPH (≥ 1000 mL) • Maternal blood transfusion • PPH (≥ 500 mL) • Therapeutic uterotonics
Pharmacological management (tranexamic acid)						
Cochrane review						
“Tranexamic acid for preventing postpartum haemorrhage” (Novikova, 2015 [59])	28 January 2015	12 RCTs	3285 women (caesarean or vaginal birth)	Trenaxamic acid	Placebo or other agents such as uterotonics	• Maternal death • Severe PPH (≥ 1000 mL) • Maternal blood transfusion • PPH (≥ 500 mL)
Non-Cochrane review						
“Anti-fibrinolytic agents in postpartum haemorrhage: a systematic review” (Ferrer, 2009 [55])	November 2008	3 RCTs	461 women (caesarean or vaginal birth)	Tranexamic acid	No treatment	• PPH (≥ 500 mL)
“Efficacy and safety of tranexamic acid administration for the prevention and/or the treatment of post-partum haemorrhage: A systematic review with meta-analysis” (Faraoni, 2014 [56])	Unclear (French)	10 RCTs	3014 women (caesarean or vaginal birth)	Tranexamic acid	Placebo	• Severe PPH (≥ 1000 mL) • PPH (≥ 500 mL) • Therapeutic uterotonics
“Prophylactic tranexamic acid in parturients at low risk for post-partum haemorrhage: Systematic review and meta-analysis” (Heesen, 2014 [58])	10 May 2013	7 RCTs	1760 women (vaginal birth)	Tranexamic acid	Placebo	• Maternal blood transfusion • PPH (≥ 500 mL)
“Does tranexamic acid prevent postpartum haemorrhage? A systematic review of randomised controlled trials” (Ker, 2016 [60])	May 2015	26 RCTs	4191 women (caesarean or vaginal birth)	Tranexamic acid	Placebo or no tranexamic acid	• Maternal death • Severe PPH (≥ 1000 mL) • Maternal blood transfusion • PPH (≥ 500 mL)
Non-pharmacological management						
Cochrane review						
“Effect of timing of umbilical cord clamping of term infants on maternal and neonatal outcomes” (McDonald, 2013 [22])	13 February 2013	15 RCTs	3911 women (vaginal birth)	Early cord clamping	Later (delayed) cord clamping	• Severe PPH (≥ 1000 mL) • Maternal blood transfusion • PPH (≥ 500 mL) • Therapeutic uterotonics
“Uterine massage for preventing postpartum haemorrhage” (Hofmeyr, 2013 [61])	30 April 2013	2 RCTs	1964 women (vaginal birth)	Uterine massage commencing after birth of the baby, before or after delivery of the placenta, or both	No intervention or a “dummy” procedure	• Maternal blood transfusion • PPH (≥ 500 mL) • Therapeutic uterotonics
“Breastfeeding or nipple stimulation for reducing postpartum haemorrhage in the third stage of labour” (Abedi, 2016 [62])	15 July 2015	4 RCTs	4608 women (vaginal birth)	Nipple stimulation	No treatment or any uterotonics	• Maternal death • Severe PPH (≥ 1000 mL) • PPH (≥ 500 mL)

### Quality of included reviews

AMSTAR [46] ratings representing the quality of systematic reviews are displayed in Table 2. The methodological quality of 16 Cochrane systematic reviews was high, with scores ranging from 10 to 11. The 10 non-Cochrane systematic review scores varied from 1 to 7. The majority of non-Cochrane reviews did not list the included and excluded studies and/or did not consider the quality of included studies.

**Table 2:** AMSTAR ratings of included systematic reviews

Review title	AMSTAR criteria											Total score (maximum of 11)
	“Apriori” design	Duplicate study selection and data extraction	Comprehensive literature search	Status of publication used as an inclusion criterion	List of studies (included and excluded) provided	Characteristics of the included studies provided	Scientific quality of the included studies assessed and documented	Scientific quality of the included studies used appropriately in formulating conclusions	Methods used to combine the findings of studies appropriate	Likelihood of publication bias assessed	Conflict of interest stated	
Active management												
Cochrane reviews												
“Fundal pressure versus controlled cord traction as part of the active management of the third stage of labour” (Peña-Martí, 2007 [49])	Yes	Yes	Yes	Yes	Yes	Yes	N/A	N/A	N/A	N/A	Yes	7
“Active versus expectant management for women in the third stage of labour” (Begley, 2015 [21])	Yes	Yes	Yes	Yes	Yes	Yes	Yes	Yes	Yes	No	Yes	10
“Controlled cord traction for the third stage of labour” (Hofmeyr, 2015 [23])	Yes	Yes	Yes	Yes	Yes	Yes	Yes	Yes	Yes	Yes	Yes	11
Non-Cochrane reviews												
“Preventing postpartum hemorrhage in low-resource settings” (McCormick, 2002 [48])	C/A	No	Yes	No	No	No	No	No	No	No	C/A	1
“Active management of the third stage of labor with and without controlled cord traction: A systematic review and meta-analysis of randomized controlled trials” (Du, 2014 [47])	C/A	Yes	Yes	No	No	Yes	Yes	Yes	Yes	No	C/A	6
Pharmacological management (oxytocin)												
Cochrane reviews												
“Prophylactic ergometrine-oxytocin versus oxytocin for the third stage of labour” (McDonald, 2004 [24])	Yes	Yes	Yes	Yes	Yes	Yes	Yes	Yes	Yes	N/A	Yes	10
“Timing of prophylactic uterotonics for the third stage of labour after vaginal birth” (Soltani, 2010 [27])	Yes	Yes	Yes	Yes	Yes	Yes	Yes	Yes	Yes	N/A	Yes	10
“Umbilical vein injection for the routine management of third stage of labour” (Mori, 2012 [25])	Yes	Yes	Yes	Yes	Yes	Yes	Yes	Yes	Yes	N/A	Yes	10
“Intramuscular versus intravenous prophylactic oxytocin for the third stage of labour” (Oladapo, 2012 [26])	Yes	Yes	Yes	Yes	Yes	Yes	N/A	N/A	N/A	N/A	Yes	7
“Carbetocin for preventing postpartum haemorrhage” (Su, 2012 [28])	Yes	Yes	Yes	Yes	Yes	Yes	Yes	Yes	Yes	Yes	Yes	11
“Prophylactic oxytocin for the third stage of labour to prevent postpartum haemorrhage” (Westhoff, 2013 [29])	Yes	Yes	Yes	Yes	Yes	Yes	Yes	Yes	Yes	Yes	Yes	11
“Oxytocin for preventing postpartum haemorrhage (PPH) in non-facility birth settings” (Pantoja, 2016 [51])	Yes	Yes	Yes	Yes	Yes	Yes	Yes	Yes	Yes	N/A	Yes	10
Non-Cochrane reviews												
“Carbetocin for the prevention of postpartum hemorrhage: a systematic review and meta-analysis of randomized controlled trials” (Jin, 2016 [50])	C/A	Yes	Yes	No	No	No	Yes	No	Yes	Yes	Yes	6
Pharmacological management (prostaglandins)												
Cochrane reviews												
“Advance misoprostol distribution for preventing and treating postpartum haemorrhage” (Oladapo, 2012 [31])	Yes	Yes	Yes	Yes	Yes	Yes	N/A	N/A	N/A	N/A	Yes	7
“Prostaglandins for preventing postpartum haemorrhage” (Tunçalp, 2012 [32])	Yes	Yes	Yes	Yes	Yes	Yes	Yes	Yes	Yes	Yes	Yes	11
“Postpartum misoprostol for preventing maternal mortality and morbidity” (Hofmeyr, 2013 [30])	Yes	Yes	Yes	Yes	Yes	Yes	Yes	Yes	Yes	Yes	Yes	11
Non-Cochrane reviews												
“Misoprostol use during the third stage of labor” (Joy, 2003 [53])	C/A	No	Yes	Yes	No	Yes	Yes	No	Yes	No	No	5
“Misoprostol in preventing postpartum hemorrhage: a meta-analysis” (Langenbach, 2006 [54])	C/A	No	Yes	No	Yes	No	Yes	No	Yes	No	No	4
“Misoprostol to prevent and treat postpartum haemorrhage: a systematic review and meta-analysis of maternal deaths and dose-related effects” (Hofmeyr, 2009 [52])	C/A	Yes	Yes	Yes	No	No	C/A	No	Yes	No	Yes	5
“Misoprostol for prevention and treatment of postpartum haemorrhage: a systematic review” (Olefile, 2013 [55])	C/A	Yes	Yes	C/A	No	Yes	Yes	No	Yes	Yes	Yes	7
Pharmacological management (ergot alkaloids)												
Cochrane reviews												
“Prophylactic use of ergot alkaloids in the third stage of labour” (Liabsuetrakul, 2007 [33])	Yes	Yes	Yes	Yes	Yes	Yes	Yes	Yes	Yes	N/A	Yes	10
Pharmacological management (tranexamic acid)												
Cochrane reviews												
“Tranexamic acid for preventing postpartum haemorrhage” (Novikova, 2015 [59])	Yes	Yes	Yes	Yes	Yes	Yes	Yes	Yes	Yes	N/A	Yes	10
Non-Cochrane reviews												
“Anti-fibrinolytic agents in postpartum haemorrhage: a systematic review” (Ferrer, 2009 [55])	C/A	Yes	Yes	Yes	No	Yes	No	Yes	Yes	No	No	6
“Efficacy and safety of tranexamic acid administration for the prevention and/or the treatment of post-partum haemorrhage: A systematic review with meta-analysis” (Faraoni, 2014 [56])	C/A	Yes	No	No	No	Yes	Yes	No	Yes	No	Yes	5
“Prophylactic tranexamic acid in parturients at low risk for post-partum haemorrhage: Systematic review and meta-analysis” (Heesen, 2014 [58])	C/A	Yes	Yes	No	No	Yes	Yes	No	Yes	No	Yes	6
“Does tranexamic acid prevent postpartum haemorrhage? A systematic review of randomised controlled trials” (Ker, 2016 [60])	Yes	No	Yes	Yes	No	Yes	Yes	Yes	No	No	Yes	7
Non-pharmacological management												
Cochrane reviews												
“Effect of timing of umbilical cord clamping of term infants on maternal and neonatal outcomes” (McDonald, 2013 [22])	Yes	Yes	Yes	Yes	Yes	Yes	Yes	Yes	Yes	Yes	Yes	11
“Uterine massage for preventing postpartum haemorrhage” (Hofmeyr, 2013 [61])	Yes	Yes	Yes	Yes	Yes	Yes	Yes	Yes	Yes	N/A	Yes	10
“Breastfeeding or nipple stimulation for reducing postpartum haemorrhage in the third stage of labour” (Abedi, 2016 [62])	Yes	Yes	Yes	Yes	Yes	Yes	Yes	Yes	Yes	N/A	Yes	10

### Quality of evidence in included reviews

Quality of evidence of the included reviews, as measured using the GRADE approach [44], varied by review and is displayed in Tables 3, 4, and 5. The risk of biased scores or imprecision was serious; therefore, the quality of evidence was low to moderate. For the rating of limitations of GRADE approach [44], we provided the result of assessing the risk of bias of the included reviews which did not provide the risk of bias for the included RCTs [48, 52–54] in Additional file 4.

**Table 3:** Maternal mortality

Intervention and comparison intervention	Anticipated absolute effects* (95% CI)		Relative effect (95% CI)	Number of participants (studies)	Quality of the evidence (GRADE)	Comments
	Risk with comparison	Risk with intervention				
Active management						
Hofmeyr 2015 [23] Active management with CCT versus without CCT	2 per 1000	2 per 1000 (1–5)	RR 1.22 (0.55–2.74)	27,300 (2 studies)	Low	Serious inconsistency, serious imprecision
Du 2014 [47] Active management with CCT versus without CCT	2 per 1000	3 per 1000 (2–4)	RR 1.55 (0.88–2.2)	23,232 (1 study)	Low	Evidence based on a single study
Oxytocin						
Pantoja, 2016 [51] Oxytocin injection versus no injection	Not estimable	Not estimable	Not estimable	1586 (1 study)	Very low	Evidence based on a single study, serious imprecision
Prostaglandin						
Hofmeyr 2009 [52] Misoprostol versus no uterotonic/placebo	Not estimable	1 per 1000 (0–2)	RR 2.0 (0.68–5.83)	22,278 (5 studies)	Moderate	Serious imprecision, reporting bias is high
Tunçalp 2012 [32] Oral misoprostol versus no uterotonic/placebo	1 per 1000	1 per 1000 (0–4)	RR 1.46 (0.24–8.81)	3965 (3 studies)	Low	Serious inconsistency
Hofmeyr 2013 [30] Misoprostol versus no uterotonic/placebo	Not estimable	1 per 1000 (0–2)	RR 2.7 (0.72–10.11)	9333 (10 studies)	Moderate	Serious imprecision
Non-pharmacological management						
Abedi, 2016 [62] Nipple stimulation versus no treatment	Not estimable	Not estimable	RR 3.03 (0.12–74.26)	4227 (1 study)	Very low	Evidence based on a single study, serious imprecision

GRADE working group grades of evidence

High quality: We are very confident that the true effect lies close to that of the estimate of the effect

Moderate quality: We are moderately confident in the effect estimate: The true effect is likely to be close to the estimate of the effect, but there is a possibility that it is substantially different

Low quality: Our confidence in the effect estimate is limited: The true effect may be substantially different from the estimate of the effect

Very low quality: We have very little confidence in the effect estimate: The true effect is likely to be substantially different from the estimate of effect

*CI* Confidence interval, *RR* risk ratio

*The risk in the intervention group (and its 95% confidence interval) is based on the assumed risk in the comparison group and the relative effect of the intervention (and its 95% CI)

**Table 4:** Blood loss greater than 1000 mL

Intervention and comparison intervention	Anticipated absolute effects* (95% CI)		Relative effect (95%CI)	Number of participants (studies)	Quality of the evidence (GRADE)	Comments
	Risk with comparison	Risk with intervention				
Active management						
McCormick 2002 [48] Active versus physiological management of third stage of labour	30 per 1000	11 per 1000 (7–17)	RR 0.36 (0.23–0.57)	4855 (3 studies)	Very low	Serious risk of bias, serious inconsistency, serious imprecision
Begley 2015 [21] Active versus expectant management of third stage of labour	24 per 1000	8 per 1000 (3–21)	RR 0.34 (0.14–0.87)	4636 (3 studies)	Very low	Serious risk of bias, serious inconsistency, serious imprecision
Du 2014 [47] Active management with CCT versus without CCT	20 per 1000	19 per 1000 (16–22)	RR 0.91 (0.77–1.08)	27,454 (3 studies)	Low	Serious risk of bias, serious inconsistency, serious imprecision
Oxytocin						
Westhoff 2013 [29] Prophylactic oxytocin versus placebo	48 per 1000	30 per 1000 (21–42)	RR 0.62 (0.44–0.87)	4162 (5 studies)	Moderate	Serious risk of bias
Soltani 2010 [27] Use of oxytocics before versus after delivery of placenta	194 per 1000	191 per 1000 (93–385)	RR 0.98 (0.48–1.98)	130 (1 study)	Low	Evidence based on a single study
Pantoja 2016 [51] Oxytocin injection in thigh versus no injection	9 per 1000	1 per 1000 (0–12)	RR 0.16 (0.02–1.30)	1569 (1 study)	Very low	Serious risk of bias, serious imprecision
Prostaglandin						
Joy 2003 [53] Oral misoprostol (400–600 mcg) versus placebo	85 per 1000	79 per 1000 (56–109)	OR 0.34 (0.64–1.33)	1505 (3 studies)	Low	Serious inconsistency, Serious imprecision
Joy 2003 [53] Rectal misoprostol (400 mcg) versus placebo	70 per 1000	48 per 1000 (24–95)	OR 0.67 (0.33–1.39)	542 (1 study)	Moderate	Evidence based on a single study
Langenbach 2006 [54] Oral or rectal misoprostol (400–600 mcg) versus placebo	83 per 1000	70 per 1000 (52–94)	RR 0.85 (0.63–1.14)	2112 (5 studies)	Very low	Serious risk of bias, serious inconsistency, serious imprecision
Hofmeyr 2009 [52] Oral or sublingual misoprostol (600 mcg) versus placebo	48 per 1000	44 per 1000 (26–75)	RR 0.92 (0.54–1.57)	4914 (5 studies)	Moderate	Serious inconsistency
Hofmeyr 2009 [52] Oral or rectal misoprostol (400 mcg) versus placebo	51 per 1000	41 per 1000 (24–70)	RR 0.80 (0.47–1.37)	3039 (5 studies)	Low	Serious inconsistency, serious imprecision
Tunçalp 2012 [32] Rectal misoprostol (400 mcg) versus placebo or no uterotonics	70 per 1000	48 per 1000 (24–96)	RR 0.69 (0.35–1.37)	542 (1 study)	Moderate	Evidence based on a single study
Tunçalp 2012 [32] Sublingual misoprostol (600 mcg) versus placebo or no uterotonics	169 per 1000	112 per 1000 (76–166)	RR 0.69 (0.35–1.37)	661 (1 study)	Moderate	Evidence based on a single study
Tunçalp 2012 [32] Buccal misoprostol (200 mcg) versus placebo or no uterotonics	123 per 1000	139 per 1000 (81–238)	RR 1.13 (0.66–1.94)	352 (1 study)	Moderate	Evidence based on a single study
Tunçalp 2012 [32] Prostaglandin versus placebo or no uterotonics	125 per 1000	69 per 1000 (27–169)	RR 0.55 (0.22–1.35)	46 (1 study)	Moderate	Evidence based on a single study
Ergot alkaloids						
Liabsuetrakul 2007 [33] Oral or intravenous ergot alkaloids versus no uterotonics	31 per 1000	10 per 1000 (1–81)	RR 0.32 (0.04–2.59)	1718 (2 studies)	Low	Serious inconsistency, serious imprecision
Tranexamic acid						
Faraoni 2014 [58] Tranexamic acids versus placebo or no treatment	96 per 1000	47 per 1000 (32–71)	RR 0.49 (0.33–0.74)	1754 (4 studies)	Moderate	Serious inconsistency
Novikova 2015 [59] Tranexamic acids versus placebo or no treatment	37 per 1000	15 per 1000 (9–27)	RR 0.40 (0.23–0.71)	2093 (6 studies)	Moderate	Serious inconsistency
Ker 2016 [60] Tranexamic acids versus placebo or no treatment	30 per 1000	13 per 1000 (6–28)	RR 0.43 (0.20–0.94)	1400 (2 studies)	Low	Serious risk of bias, serious imprecision
Timing of cord clamping						
McDonald, 2013 [22] Early versus late cord clamping	34 per 1000	35 per 1000(22–56)	RR 1.04 (0.65–1.65)	2066 (5 studies)	Moderate	Serious inconsistency
Uterine massage						
Hofmeyr 2013 [61] Uterine massage versus no massage	2 per 1000	5 per 1000 (0–44)	RR 2.96 (0.31–28.35)	1291 (2 studies)	Low	Serious inconsistency, serious imprecision

GRADE working group grades of evidence

High quality: We are very confident that the true effect lies close to that of the estimate of the effect

Moderate quality: We are moderately confident in the effect estimate: the true effect is likely to be close to the estimate of the effect, but there is a possibility that it is substantially different

Low quality: Our confidence in the effect estimate is limited: the true effect may be substantially different from the estimate of the effect

Very low quality: We have very little confidence in the effect estimate: the true effect is likely to be substantially different from the estimate of effect

*CI* Confidence interval, *RR* risk ratio

*The risk in the intervention group (and its 95% confidence interval) is based on the assumed risk in the comparison group and the relative effect of the intervention (and its 95% CI)

**Table 5:** Blood transfusion

Intervention and comparison intervention	Anticipated absolute effects* (95% CI)		Relative effect (95% CI)	Number of participants (studies)	Quality of the evidence (GRADE)	Comments
	Risk with comparison	Risk with intervention				
Active management						
McCormick 2002 [48] Active versus physiological management of third stage of labour	30 per 1000	9 per 1000 (6 to 15)	RR 0.32 (0.20–0.51)	4855 (3 studies)	Low	Serious risk of bias, serious imprecision
Begley 2015 [21] Active versus expectant management of third stage of labour	29 per 1000	10 per 1000 (6 to 16)	RR 0.35 (0.22–0.55)	4829 (4 studies)	Moderate	Serious imprecision
Du 2014 [47] Active management with CCT versus without CCT	5 per 1000	5 per 1000 (3 to 7)	RR 0.96 (0.69–1.33)	28,062 (3 studies)	Moderate	Serious risk of bias
Hofmeyr 2015 [23] Active management with CCT versus without CCT	5 per 1000	5 per 1000 (3 to 7)	RR 0.94 (0.68–1.32)	27,662 (2 studies)	High	
Oxytocin						
Westhoff 2013 [29] Oxytocin versus placebo	12 per 1000	10 per 1000 (5 to 21)	RR 0.89 (0.44–1.78)	3120 (3 studies)	Moderate	Serious imprecision
Soltani 2010 [27] Use of oxytocics before versus after delivery of placenta	7 per 1000	6 per 1000 (2 to 20)	RR 0.79 (0.23–2.73)	1667 (3 studies)	Moderate	Serious imprecision
Mori 2012 [25] Umbilical vein injection of a saline versus oxytocin with a saline alone	Not estimable	Not estimable	RR 3.32 (0.14–78.97)	78 (1 study)	Low	Serious inconsistency, serious imprecision
Prostaglandin						
Tunçalp 2012 [32] Oral misoprostol (400–600 mcg) versus placebo or no uterotonics	7 per 1000	2 per 1000 (1 to 6)	RR 0.31 (0.10–0.94)	3519 (5 studies)	Moderate	Serious imprecision
Tunçalp 2012 [32] Buccal misoprostol (200 mcg) versus placebo or no uterotonics	16 per 1000	11 per 1000 (4 to 30)	RR 0.68 (0.24–1.89)	1108 (2 studies)	Moderate	Serious imprecision
Olefile 2013 [55] Oral misoprostol (600 mcg) versus placebo	9 per 1000	1 per 1000 (0 to 10)	RR 0.14 (0.02–1.15)	1620 (1 study)	Moderate	Evidence based on a single study
Ergot alkaloids						
Liabsuetrakul 2007 [33] Oral or intravenous ergot alkaloids versus no uterotonics	7 per 1000	2 per 1000 (1 to 9)	RR 0.33 (0.08–1.40)	1868 (3 studies)	Low	Serious risk of bias, serious imprecision
Tranexamic acid						
Heesen 2014 [58] Tranexamic acids versus placebo	53 per 1000	18 per 1000 (11 to 32)	RR 0.34 (0.20–0.60)	1662 (6 studies)	Moderate	Serious imprecision
Novikova 2015 [59] Tranexamic acids versus placebo or no treatment	31 per 1000	7 per 1000 (3 to 16)	RR 0.24 (0.11–0.53)	1698 (6 studies)	Moderate	Serious imprecision
Ker 2016 [60] Tranexamic acids versus placebo or no treatment	44 per 1000	14 per 1000 (8 to 23)	RR 0.31 (0.18–0.53)	2272 (9 studies)	Low	Serious risk of bias, serious imprecision
Timing of cord clamping						
McDonald, 2013 [22] Early versus late cord clamping	15 per 1000	15 per 1000 (7 to 35)	RR 1.02 (0.44–2.37)	1345 (3 studies)	Moderate	Serious imprecision
Uterine massage						
Hofmeyr 2013 [61] Uterine massage before placental delivery versus no massage	6 per 1000	6 per 1000 (2 to 23)	RR 0.97 (0.26–3.58)	1257 (2 studies)	Low	Serious inconsistency, serious imprecision
Hofmeyr 2013 [61] Uterine massage before and after placental delivery versus no massage	6 per 1000	5 per 1000 (1 to 20)	RR 0.97 (0.26–3.58)	1457 (3 studies)	Low	Serious inconsistency, serious imprecision

GRADE working group grades of evidence

High quality: We are very confident that the true effect lies close to that of the estimate of the effect

Moderate quality: We are moderately confident in the effect estimate: the true effect is likely to be close to the estimate of the effect, but there is a possibility that it is substantially different

Low quality: Our confidence in the effect estimate is limited: The true effect may be substantially different from the estimate of the effect

Very low quality: We have very little confidence in the effect estimate: the true effect is likely to be substantially different from the estimate of effect

*CI* Confidence interval, *RR* risk ratio

*The risk in the intervention group (and its 95% confidence interval) is based on the assumed risk in the comparison group and the relative effect of the intervention (and its 95% CI)

### Effect of interventions for maternal mortality

We identified seven reviews that examined maternal mortality: two regarding active management of the third stage of labour [23, 47], one examining the use of oxytocin [51], three examining the use of prostaglandins [30, 32, 52], and one examining nipple stimulation [62]. Table 3 portrays the effects of these management techniques.

#### Active management versus contrasting management

In the comparison of active management during the third stage of labour with and without controlled cord traction, there was no significant difference between the groups: (31/11,616 versus 20/11,616; relative risk (RR) = 1.55, 95% confidence interval (CI) = 0.88 to 2.72; 1 RCT; 23,232 women) [47] and (34/13,650 versus 25/13,650; RR = 1.22, 95% CI = 0.55 to 2.74; 2 RCTs; 27,300 women; heterogeneity; *I*^2^ = 32%) [23].

#### Oxytocin versus no treatment

There were no cases reporting maternal deaths related to oxytocin injection. Therefore, the effect of an oxytocin injection (10 IU) in the thigh compared to no treatment could not be estimated [51].

#### Prostaglandin versus placebo

In the comparison of misoprostol (> 600 μg) versus placebo, there was no difference between the groups (8/11,153 versus 3/11,125; RR = 2.0, 95% CI = 0.68 to 5.83; 5 RCTs; 22,278 women; heterogeneity; *I*^2^ = 0%) [52] and (6/4646 versus 1/4707; RR = 2.70, 95% CI = 0.72 to 10.11; 10 RCTs; 9333 women; heterogeneity; *I*^2^ = 0%) [30]. There was also no difference between the groups comparing oral misoprostol (600 μg) with placebo or no uterotonic use (2/1975 versus 1/1990; RR = 1.46, 95% CI = 0.24 to 8.81; 3 RCTs; 3965 women; heterogeneity; *I*^2^ = 29%) [32].

#### Nipple stimulation versus no treatment

In the comparison of breastfeeding immediately after delivery versus no breastfeeding or nipple stimulation, no significant difference was found between the groups (1/2104 versus 0/2123; RR = 3.03, 95% CI = 0.12 to 74.26; 1 RCT; 4227 women) [62].

### Effect of interventions for blood loss greater than 1000 mL

A total of 20 reviews examining blood loss > 1000 mL were identified: four regarding active management of the third stage of labour [21, 23, 47, 48], six examining the use of oxytocin [24, 27–29, 50, 51], four examining the use of prostaglandins [32, 52–54], one examining the use of ergot alkaloids [33], three examining the use of tranexamic acid [56, 59, 60], one examining the timing of clamping umbilical cord [22], and one examining uterine massage [61]. Table 4 only displays interventions that compared the intervention with placebo or contrasting management.

#### Active management versus contrasting management

Compared to physiological or expectant management, active management resulted in a significant reduction in severe PPH (26/2421 versus 72/2434; RR = 0.36, 95% CI = 0.23 to 0.57; 3 RCTs; 4855 women; heterogeneity; *I*^2^ = 55%) [48] and (21/2299 versus 57/2337; average RR = 0.34, 95% CI = 0.14 to 0.87; 3 RCTs; 4636 women; heterogeneity; *I*^2^ = 60%) [21]. For the comparison of active management in the third stage of labour with and without controlled cord traction, there was no significant difference between the groups (256/13,727 versus 281/13,727; RR = 0.91, 95% CI = 0.77 to 1.08; 3 RCTs; 27,454 women; heterogeneity; *I*^2^ = 0%) [47] and (256/13,727 versus 281/13,727; RR = 0.91, 95% CI = 0.77 to 1.08; 3 RCTs; 27,454 women; heterogeneity; *I*^2^ = 0%) [23].

#### Oxytocin versus placebo

Prophylactic use of oxytocin showed a significant reduction in severe PPH compared to a placebo (52/2367 versus 87/1795; RR = 0.62, 95% CI = 0.44 to 0.87; 5 RCTs; 4162 women; heterogeneity; *I*^2^ = 0%) [29]. Administration of oxytocin before versus that after delivery of the placenta did not significantly alter the incidence of severe PPH (11/58 versus 14/72; RR = 0.98, 95% CI = 0.48 to 1.98; 1 RCT; 130 women) [27]. Prophylactic oxytocin injection (10 IU) in the thigh in non-facility settings versus no injection resulted in no significant difference between the groups (8/682 versus 1/888; RR = 0.16, 95% CI = 0.02 to 1.30; 1 RCT; 1570 women) [51].

#### Prostaglandin versus placebo

There was no difference between the groups comparing the routes of administration of misoprostol: oral misoprostol (400 to 600 μg) with placebo (58/736 versus 65/769; RR = 0.92, 95% CI = 0.64 to 1.33; 3 RCTs; 1505 women; heterogeneity; *I*^2^ = 28%) [53], rectal misoprostol (400 μg) with placebo (13/270 versus 19/272; RR = 0.67, 95% CI = 0.33 to 1.39; 1 RCT; 542 women) [53], misoprostol (oral or rectal 400 to 600 μg) with placebo (73/1037 versus 89/1075; RR = 0.85, 95% CI = 0.63 to 1.14; 5 RCTs; 2112 women; heterogeneity; *I*^2^ = 2%) [54], oral or sublingual misoprostol (600 μg) with placebo (101/2457 versus 118/2457; RR = 0.92, 95% CI = 0.54 to 1.57; 6 RCTs; 4914 women; heterogeneity; *I*^2^ = 66%) [52], oral or rectal misoprostol (400 μg) with placebo (61/1526 versus 77/1513; RR = 0.80, 95% CI = 0.47 to 1.37; 5 RCTs; 3039 women; heterogeneity; *I*^2^ = 58%) [52], rectal misoprostol (400 μg) with placebo or no uterotonics (13/270 versus 19/272; RR = 0.69, 95% CI = 0.35 to 1.37; 1 RCT; 542 women) [32], sublingual misoprostol (600 μg) with placebo or no uterotonics (0/330 versus 0/331; RR = 0.66, 95% CI = 0.45 to 0.98; 1 RCT; 661 women) [32], buccal misoprostol (200 μg) with placebo or no uterotonics (24/173 versus 22/179; RR = 1.13, 95% CI 0.66 to 1.94; 1 RCT; 352 women) [32], and intramuscular prostaglandin with placebo or no uterotonics (5/22 versus 10/24; RR = 0.55, 95% CI = 0.22 to 1.35; 1 RCT; 46 women) [32].

#### Ergot alkaloids versus placebo

There was no significant difference between the groups comparing oral or intravenous ergot alkaloids with no uterotonics (13/851 versus 27/867; RR = 0.32, 95% CI = 0.04 to 2.59; 2 RCTs; 1718 women; heterogeneity; *I*^2^ = 74%) [33].

#### Tranexamic acid versus placebo

Tranexamic acid significantly reduced the incidence of severe PPH compared to placebo or no treatment (50/882 versus 84/872; RR = 0.49, 95% CI = 0.33 to 0.74; 4 RCTs; 1754 women; heterogeneity; *I*^2^ = 0%) [56], (16/1051 versus 39/1042; RR = 0.40, 95% CI = 0.23 to 0.71; 6 RCTs; 2093 women; heterogeneity; *I*^2^ = 0%) [59] and (9/703 versus 21/697; RR = 0.43; 95% CI = 0.20 to 0.94; 2 RCTs; 1400 women; heterogeneity; *I*^2^ = 0%) [60].

#### Early versus late cord clamping

There was no significant difference between the groups for early cord clamping compared to late clamping (34/975 versus 37/1091; RR = 1.04, 95% CI = 0.65 to 1.65; 5 RCTs; 2066 women; heterogeneity; I^2^ = 0%) [22].

#### Uterine massage versus no uterine massage

There was no significant difference between groups implementing uterine massage prior to placental delivery versus none (3/652 versus 1/639; RR = 2.96, 95% CI = 0.31 to 28.35; 2 RCTs; 1,291women; heterogeneity; *I*^2^ = not applicable) [61].

### Effect of interventions using blood transfusion

We included 18 reviews with blood transfusion as an intervention: four examining active management of the third stage of labour [21, 23, 47, 48], six examining oxytocin use [24, 25, 27–29, 50], two examining prostaglandins [32, 55], one examining ergot alkaloids [33], three examining tranexamic acid [58–60], one examining cord clamping timing [22], and one examining uterine massage [61]. Table 5 displays the effect of interventions only compared with placebo or contrasting management.

#### Active management versus contrasting management

Active management of the third stage of labour significantly reduced the use of blood transfusions compared to physiological management or expectant management (23/2421 versus 72/2434; RR = 0.32, 95% CI = 0.20 to 0.51; 3 RCTs; 4855 women; heterogeneity; *I*^2^ = 80%) [48] and (24/2402 versus 71/2427; RR = 0.35, 95% CI = 0.22 to 0.55; 4 RCTs; 4829 women; heterogeneity; *I*^2^ = 0%) [21]. There was no significant difference between the groups for active management, with versus without controlled cord traction: (68/14,024 versus 71/14,038; RR = 0.96, 95% CI = 0.69 to 1.33; 3 RCTs; 28,062 women; heterogeneity; *I*^2^ = 0%) [47] and (67/13,824 versus 71/13,838; RR = 0.94, 95% CI = 0.68 to 1.32; 2 RCTs; 27,662 women; heterogeneity; *I*^2^ = 0%) [23].

#### Oxytocin versus placebo

There was no significant difference between groups that compared oxytocin with placebo (17/1848 versus 15/1272; RR = 0.89, 95% CI = 0.44 to 1.78; 3 RCTs; 3120 women; heterogeneity; *I*^2^ = 0%) [29], administration of oxytocin before versus after delivery of the placenta (4/830 versus 6/837; RR = 0.79, 95% CI = 0.23 to 2.73; 3 RCTs; 1667 women; heterogeneity; *I*^2^ = 0%) [27], and umbilical vein injection of saline plus oxytocin versus saline injection alone (1/37 versus 0/41; RR = 3.32, 95% CI = 0.14 to 78.97; 1 RCT; 78 women) [25].

#### Prostaglandin versus placebo

Oral misoprostol (400–600 μg) significantly reduced the need for blood transfusion compared to placebo or no uterotonics (3/1761 versus 12/1758; RR = 0.31, 95% CI = 0.10 to 0.94; 5 RCTs; 3519 women; heterogeneity; *I*^2^ = 0%) [32]. There was no significant difference between the groups that compared buccal misoprostol (200 μg) with placebo or no uterotonics (6/550 versus 9/558; RR = 0.68, 95% CI = 0.24 to 1.89; 2 RCTs; 1108 women; heterogeneity; *I*^2^ = 0%) [32] and that compared oral misoprostol (600 μg) with placebo (1/812 versus 7/808; RR = 0.14, 95% CI = 0.02 to 1.15; 1 RCT; 1620 women) [55].

#### Ergot alkaloids versus placebo

There was no statistical difference in the need for blood transfusion between the ergot alkaloid and no uterotonics groups (2/951 versus 6/917; RR = 0.33, 95% CI = 0.08 to 1.40; 3 RCTs; 1868 women; heterogeneity; *I*^2^ = 0%) [33].

#### Tranexamic acid versus placebo

Tranexamic acid significantly reduced the incidence of blood transfusion compared to placebo: (15/838 versus 44/824; RR = 0.34, 95% CI = 0.20 to 0.60; 6 RCTs; 1662 women; heterogeneity; *I*^2^ = 0%) [58], (6/855 versus 26/843; RR = 0.24, 95% CI = 0.11 to 0.53; 6 RCTs; 1698 women; heterogeneity; *I*^2^ = 0%) [59], and (14/1143 versus 50/1129; RR = 0.31, 95% CI = 0.18 to 0.53; 9 RCTs; 2272 women; heterogeneity; *I*^2^ = 0%) [60].

#### Early versus late cord clamping

No difference was demonstrated in the need for blood transfusion when early and late cord clamping were compared (10/669 versus 10/676; RR = 1.02, 95% CI = 0.44 to 2.37; 3 RCTs; 1345 women; heterogeneity; *I*^2^ = 0%) [22].

#### Uterine massage versus no uterine massage

There was no significant difference between groups comparing uterine massage before placental delivery with no massage (4/637 versus 4/620; RR = 0.97, 95% CI = 0.26 to 3.58; 2 RCTs; 1257 women; heterogeneity; *I*^2^ = 42%) [61] and comparing uterine massage before and after placental delivery with no massage (4/735 versus 4/722; RR = 0.97, 95% CI = 0.26 to 3.58; 3 RCTs; 1457 women; heterogeneity; *I*^2^ = 42%) [61].

## Discussion

### Summary of main results

In this overview, we appraised and summarized the evidence from 29 systematic reviews that assessed the effectiveness of prophylactic management of PPH in the third stage of labour, including caesarean sections. We then summarized review results based on three outcomes: (1) maternal mortality, (2) blood loss greater than 1000 mL, and (3) use of blood transfusion.

#### Maternal mortality

There were no effective interventions which we identified. Active management of the third stage of labour with or without controlled cord traction [23, 47], nipple stimulation [62], and prostaglandins [30, 32, 52] were the only interventions that assessed this outcome.

#### Blood loss greater than 1000 mL

A few effective interventions were identified: active management of the third stage of labour rather than physiological or expectant management [21, 48], administration of oxytocin (5–10 IU) [29], and tranexamic acid versus placebo [56, 59, 60]. A lack of evidence was identified (i.e., no studies found) for the following interventions: fundal pressure versus controlled cord traction as part of the active management of the third stage of labour [49] and intramuscular versus intravenous oxytocin [26].

#### Use of blood transfusion

The following were effective interventions resulting in reduced blood transfusions: active management of the third stage of labour rather than physiological management [48], active management of the third stage of labour rather than expectant management [21], oral misoprostol (400–600 μg) compared to placebo [32], and tranexamic acid compared to placebo [58–60].

### Overall completeness and applicability of evidence

This overview systematically summarized 29 systematic reviews of eight different methods that included pharmacological and non-pharmacological managements; however, this overview had several limitations. We only focused on interventions that compared placebo or contrasting management. In order to make results applicable to practice, broad-based pharmacological interventions compared to other pharmacological interventions should have been included in our main analysis. Maternal mortality was reported in a few reviews [23, 30, 32, 47, 52, 62] (Table 3). Because PPH is one of the leading causes of maternal death, a greater number of reviews examining various interventions are needed to apply findings to clinical settings. Other outcomes had large amounts of data from numerous trials, but most reviews had serious flaws due to high heterogeneity or few outcome events (Tables 4 and 5). This review did not perform sub-group analysis for the delivery mode or setting. Study participants in the included reviews had undergone vaginal delivery or caesarean section, yet none of the reviews identified risk to participants or examined labour interventions. In this overview, we did not show participant details, settings, or interventions. As such, clinicians who wish to apply the evidence from this review to clinical settings should do so cautiously.

### Quality of the evidence

Using the AMSTAR tool [46], we found that the quality of the Cochrane systematic reviews was high; however, non-Cochrane systematic reviews varied from low to high. Only three of 11 non-Cochrane systematic reviews provided the list of included and excluded studies. Providing the list of all studies which appear to meet the inclusion criteria could reduce the risk of publication bias. Most of the systematic reviews in which the quality of evidence was low needed to include a comprehensive research analysis and should have provided details about publication bias.

### Potential biases in the overview process

For this overview, we adopted the method outlined in the Cochrane systematic reviews of interventions [39], which minimized the potential bias introduced at all stages in the review process.

### Agreements and disagreements with other studies or reviews

WHO has published guidelines for the prevention and treatment of PPH [5]. This document assessed numerous systematic reviews and RCTs that were included in the present overview. The intrapartum guideline, which was published by the National Institute for Health and Care Excellence, assessed active management of the third stage of labour [18]. Several RCTs were included in the NICE guideline despite the omission of a Cochrane systematic review. However, in this overview, we were able to include a broader spectrum of published systematic reviews.

## Conclusions

No effective prophylactic management of maternal mortality was identified. Most methods of effective prophylactic management of PPH were supported by evidence; however, they were limited to low- or moderate-quality evidence. Higher quality studies are therefore needed. Study participants had undergone vaginal delivery or caesarean section, and their risks and presence or absence of labour interventions were unclear. Therefore, when these prophylactic strategies are used, the state of participants and access to medical care should be considered.

The critical outcome measures about prevention and treatment of PPH are proposed in the WHO guidelines [5]. However, the systematic reviews assessed herein had a variety of outcome measures, as did the individual trials, reducing our ability to compare the results. For example, the outcome regarding the proportion of PPH had several cut-points (including ≥ 300, 400, and 500 mL). This made it difficult to pool results and show all the evidence from similar trials. It is therefore recommended that trials examining preventive interventions for PPH use consistent outcome measures and those that are recommended in the WHO guidelines [5].

## Additional files

Additional file 1: PRISMA 2009 Checklist. (DOC 68 kb)

Additional file 2: Search strategies for included reviews. (DOCX 22 kb)

Additional file 3: References to studies excluded from this review. (DOCX 16 kb)

Additional file 4: Risk of bias of reviews [48, 52–54]. (DOCX 88 kb)

## Abbreviations

AMSTAR: A measurement tool to assess reviews
EMBASE: Excerpta Medica database
GRADE: Grades of Recommendation Assessment, Development and Evaluation
MEDLINE: Medical Literature Analysis and Retrieval System Online abbreviations
NICE: National Institute for Health and Care Excellence
PPH: Postpartum haemorrhage
PRISMA: Preferred reporting items for systematic reviews and meta-analyses
WHO: The World Health Organization
