# Supplementary material for: Prophylactic management of postpartum haemorrhage in the third stage of labour: an overview of systematic reviews
Source: Syst Rev. 2018 Oct 11;7:156. doi: 10.1186/s13643-018-0817-3 (PMC6180398; doi:10.1186/s13643-018-0817-3)
Supplement: Supplementary file 2 — Search strategies for included reviews. (DOCX 22 kb) [file 13643_2018_817_MOESM2_ESM.docx]

Additional file 2　Search Strategies (October 11, 2016)

【Cochrane Database of Systematic Reviews : Issue 10 of 12, October 2016】

#1 MeSH descriptor: [Postpartum Hemorrhage] explode all trees and with qualifier(s): [Prevention & control - PC] 20

【Database of Abstracts of Reviews of Effect : Issue 2 of 4, April 2015】

#1 MeSH descriptor: [Postpartum Hemorrhage] explode all trees and with qualifier(s): [Prevention & control - PC] 13

【MEDLINE (via EBSCO) (1966 to 11 October 2016)】

#1 PT (review or review,tutorial or review, academic) 2,157,759

#2 TI (medline or medlars or embase or pubmed or cochrane) or AB (medline or medlars or embase or pubmed or cochrane) 127,495

#3 TI (scisearch or psychinfo or psycinfo) or AB (scisearch or psychinfo or psycinfo)  13,893

#4  TI (psychlit or psyclit) or AB (psychlit or psyclit) 891

#5  TI cinahl or AB cinahl 15,228

#6  TI ((hand N2 search*) or (manual* N2 search*)) or AB ((hand N2 search*) or (manual* N2 search*)) 9,677

#7 TI (electronic database* or bibliographic database* or computeri?ed database* or online database*) or AB (electronic database* or bibliographic database* or computeri?ed database* or online database*)  25,702

#8 TI (pooling or pooled or mantel haenszel) or AB (pooling or pooled or mantel haenszel) 70,467

#9 TI (peto or dersimonian or der simonian or fixed effect) or AB (peto or dersimonian or dersimonian or fixed effect)  5,904

#10  PT (retraction of publication or retracted publication) 9,201

#11 #2 OR #3 OR #4 OR #5 OR #6 OR #7 OR #8 OR #9 OR #10 212,673

#12 #1 AND #11  101,378

#13 PT meta-analysis  68,352

#14  TI (meta-analys* or meta analys* or metaanalys*) or AB (meta-analys* or meta analys* or metaanalys*) 97,129

#15 TI (systematic* N5 review*) or AB (systematic* N5 review*) 98,226

#16  TI (systematic* N5 overview*) or AB (systematic* N5 overview*) 1,403

#17 TI (quantitativ* N5 review*) or AB (quantitativ* N5 review*) 3,857

#18 TI (quantitativ* N5 overview*) or AB (quantitativ* N5 overview*) 271

#19 TI (quantitativ* N5 synthesis*) or AB (quantitativ* N5 synthesis*) 1,987

#20 TI (methodologic* N5 review*) or AB (methodologic* N5 review*) 4,962

#21 TI (methodologic* N5 overview*) or AB (methodologic* N5 overview*)  328

#22 TI (integrative research review* or research integration) or AB (integrative research review* or research integration)  1,016

#23 #13 OR #14 OR #15 OR #16 OR #17 OR #18 OR #19 OR #20 OR #21 OR #22 183,051

#24  #12 OR #23 225,312

#25 (MH "Postpartum Hemorrhage")  5,630

#26 #24 AND #25 151

【EMBASE (1966 to 11 October 2016)】

#1  **'review'**/exp 3,282,477

#2  (**literature** NEAR/3 **review***):ab,ti 260,154

#3  **meta** AND **'analysis'**/exp 158,171

#4  **'systematic review'**/exp 118,034

#5  **#1** OR **#2** OR **#3** OR **#4** 3,361,778

#6  **medline**:ab,ti OR **medlars**:ab,ti OR **embase**:ab,ti OR **pubmed**:ab,ti OR **cinahl**:ab,ti OR **amed**:ab,ti OR **psychlit**:ab,ti OR **psyclit**:ab,ti OR**psychinfo**:ab,ti OR **psycinfo**:ab,ti OR **scisearch**:ab,ti OR **cochrane**:ab,ti 65,544

#7  **retracted** AND **article** 10,945

#8  **#6** OR **#7** 76,439

#9  **#5** AND **#8** 60,810

#10  (**systematic*** NEAR/2 (**review*** OR **overview**)):ab,ti 117,115

#11  **meta?anal*** 5

#12  **meta** AND **anal*** 189,354

#13  **'meta analysis'** 170,903

#14  **metaanal*** 7,386

#15  **metanal*** 648

#16  **#11** OR **#12** OR **#13** OR **#14** OR **#15** 190,908

#17  **#9** OR **#10** OR **#16** 272,470

#18  **'postpartum hemorrhage'**/exp/mj 4,345

#19  **#17** AND **#18** 139

#20  **#17** AND **#18** AND [embase]/lim 107
