# Supplementary material for: Prophylactic management of postpartum haemorrhage in the third stage of labour: an overview of systematic reviews
Source: Syst Rev. 2018 Oct 11;7:156. doi: 10.1186/s13643-018-0817-3 (PMC6180398; doi:10.1186/s13643-018-0817-3)
Supplement: Supplementary file 3 — References to studies excluded from this review. (DOCX 16 kb) [file 13643_2018_817_MOESM3_ESM.docx]

**References to studies excluded from this review**

1. Calvert C, Thomas SL, Ronsmans C, Wagner KS, Adler AJ, Filippi V. Identifying regional variation in the prevalence of postpartum haemorrhage: a systematic review and meta-analysis. PLoS One 2012;7:e41114.
2. Chong Y, Su L, Arulkumaran S. Current strategies for the prevention of postpartum haemorrhage in the third stage of labour. Curr Opin Obstet Gynecol 2004;16(2):143-50.
3. Elbourne DR, Prendiville WJ, Carroli G, Wood J, McDonald S. Prophylactic use of oxytocin in the third stage of labour. Cochrane Database Syst Rev 2001(4):CD001808.
4. Franchini M, Franchi M, Bergamini V, Salvagno GL, Montagnana M, Lippi G. A critical review on the use of recombinant factor VIIa in life-threatening obstetric postpartum hemorrhage. Semin Thromb Hemost 2008;34(1):104-12.
5. Garofalo M, Abenhaim HA. Early versus delayed cord clamping in term and preterm births: a review. J Obstet Gynaecol Can 2012;34(6):525-31.
6. Gizzo S, Patrelli TS, Gangi SD, Carrozzini M, Saccardi C, Zambon A, et al. Which uterotonic is better to prevent the postpartum hemorrhage? Latest news in terms of clinical efficacy, side effects, and contraindications: a systematic review. Reprod Sci 2013;20(9):1011-9.
7. Gülmezoglu ,A.M., Forna F, Villar J, Hofmeyr GJ. Prostaglandins for prevention of postpartum haemorrhage. Cochrane Database Syst Rev 2001(4):CD000494.
8. Hundley VA, Avan BI, Sullivan CJ, Graham WJ. Should oral misoprostol be used to prevent postpartum haemorrhage in home-birth settings in low-resource countries? A systematic review of the evidence. BJOG 2013;120(3):277-85.
9. Leduc D, Senikas V, Lalonde AB, Ballerman C, Biringer A, Delaney M, et al. Active management of the third stage of labour: prevention and treatment of postpartum hemorrhage. J Obstet Gynaecol Can 2009;31(10):980-93.
10. Mategrano VA, Gabay MP. Misoprostol in the prevention of postpartum hemorrhage. Ann Pharmacother 2001;35(12):1648-52.
11. Prata N, Bell S, Weidert K. Prevention of postpartum hemorrhage in low-resource settings: current perspectives. Int J Womens Health. 2013;5:737-52.
12. Prendiville W, Elbourne D, Chalmers I. The effects of routine oxytocic administration in the management of the third stage of labour: an overview of the evidence from controlled trials. Br J Obstet Gynaecol 1988;95(1):3-16.
13. Prendiville WJ, Elbourne D, McDonald S. Active versus expectant management in the third stage of labour. Cochrane Database Syst Rev 2000(2):CD000007.
14. Roach MK, Abramovici A, Tita ATN. Dose and duration of oxytocin to prevent postpartum hemorrhage: a review. Am J Perinatol 2013;30(7):523-8.
15. Su LL, Chong YS, Samuel M. Oxytocin agonists for preventing postpartum haemorrhage. Cochrane Database Syst Rev 2007 (3):CD005457.
16. Tsu VD, Langer A, Aldrich T. Postpartum hemorrhage in developing countries: is the public health community using the right tools? Int J Gynaecol Obstet 2004;85 Suppl 1:S42-S51.
17. Villar J, Gülmezoglu ,A.M., Hofmeyr GJ, Forna F. Systematic review of randomized controlled trials of misoprostol to prevent postpartum hemorrhage. Obstet Gynecol 2002;100(6):1301-12.
