# Supplementary figures and images for: Prophylactic management of postpartum haemorrhage in the third stage of labour: an overview of systematic reviews
Source: Syst Rev. 2018 Oct 11;7:156. doi: 10.1186/s13643-018-0817-3 (PMC6180398; doi:10.1186/s13643-018-0817-3)

Risk of bias summary of the systematic reviews [48, 52-54]

McCormick, 2002 [48]


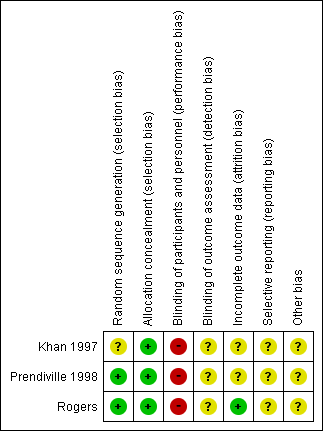


Hofmeyr, 2009 [52]


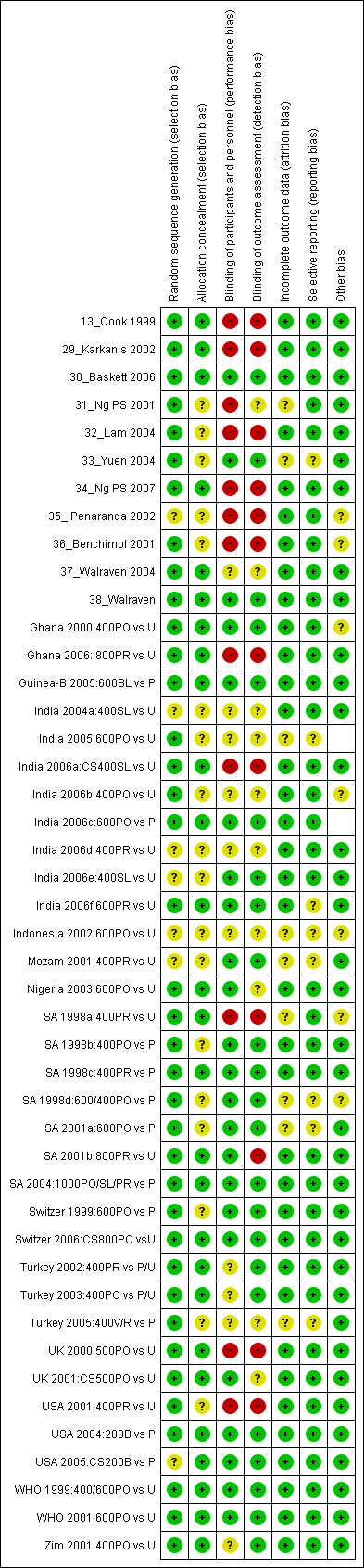


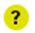

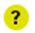


Joy, 2003 [53]


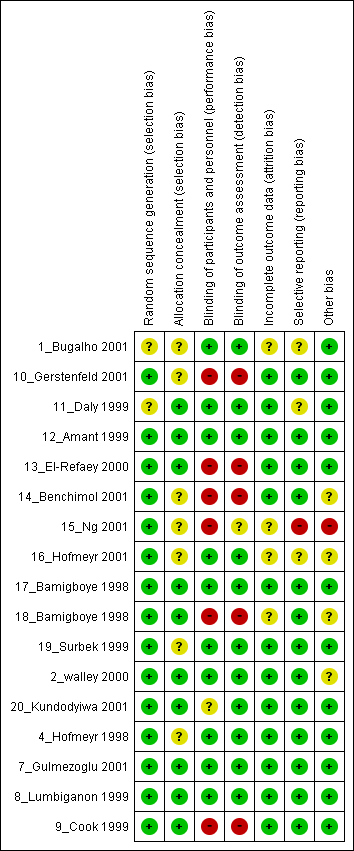


Langenbach, 2005 [54]


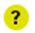

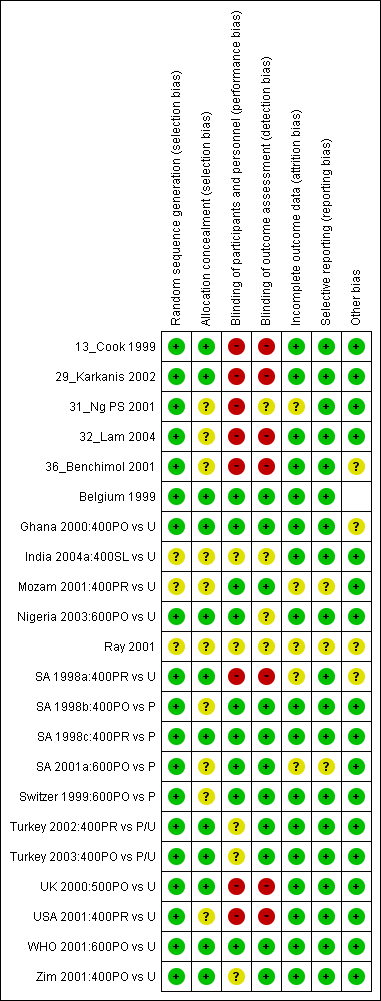

Supplement: Supplementary file 4 — Risk of bias of reviews [48, 52–54]. (DOCX 88 kb) [file 13643_2018_817_MOESM4_ESM.docx]
